# Supplementary material for: Structures of human SGLT in the occluded state reveal conformational changes during sugar transport
Source: Nat Commun. 2023 May 22;14:2920. doi: 10.1038/s41467-023-38720-1 (PMC10203128; doi:10.1038/s41467-023-38720-1)
Supplement: Supplementary file 1 — Supplementary information [file 41467_2023_38720_MOESM1_ESM.pdf]

Supplementary Information for

Structures of human SGLT in the occluded state reveal conformational changes during sugar transport

Wenhao Cui, Yange Niu, Zejian Sun, Rui Liu, and Lei Chen*

* To whom correspondence could be addressed: Lei Chen (chenlei2016@pku.edu.cn)

This PDF file contains:

Supplementary Figs. 1-9

Supplementary Table 1

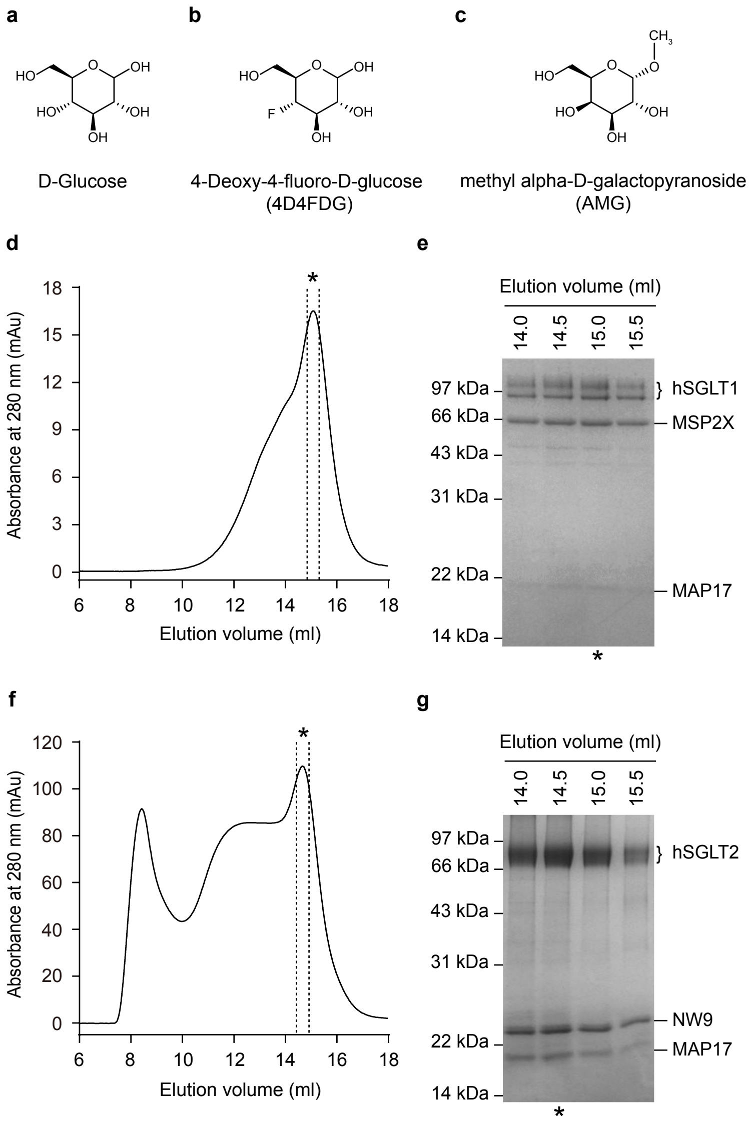

Supplementary Fig. 1 | Purification of the hSGLT1-Map17 complex and the hSGLT2-MAP17 complex. a, The chemical structure of D-glucose. **b,** The chemical structure of 4D4FDG. **c,** The chemical structure of AMG. **d,** The gel filtration profile of hSGLT1 in nanodisc. Fractions between dashed lines indicated by asterisk were used for cryo-EM sample preparation. **e**, SDS-PAGE analysis of the purified hSGLT1 in nanodisc. Asterisk indicates the fraction for cryo-EM sample preparation. **f**, The gel filtration profile of hSGLT2 in nanodisc. Fractions between dashed lines indicated by asterisk were used for cryo-EM sample preparation. **g**, SDS-PAGE analysis of the purified hSGLT2 in nanodiscs. Asterisk indicates the fraction for cryo-EM sample preparation. Experiment was repeated independently more than three times with similar results. Source data are provided as a Source Data file.

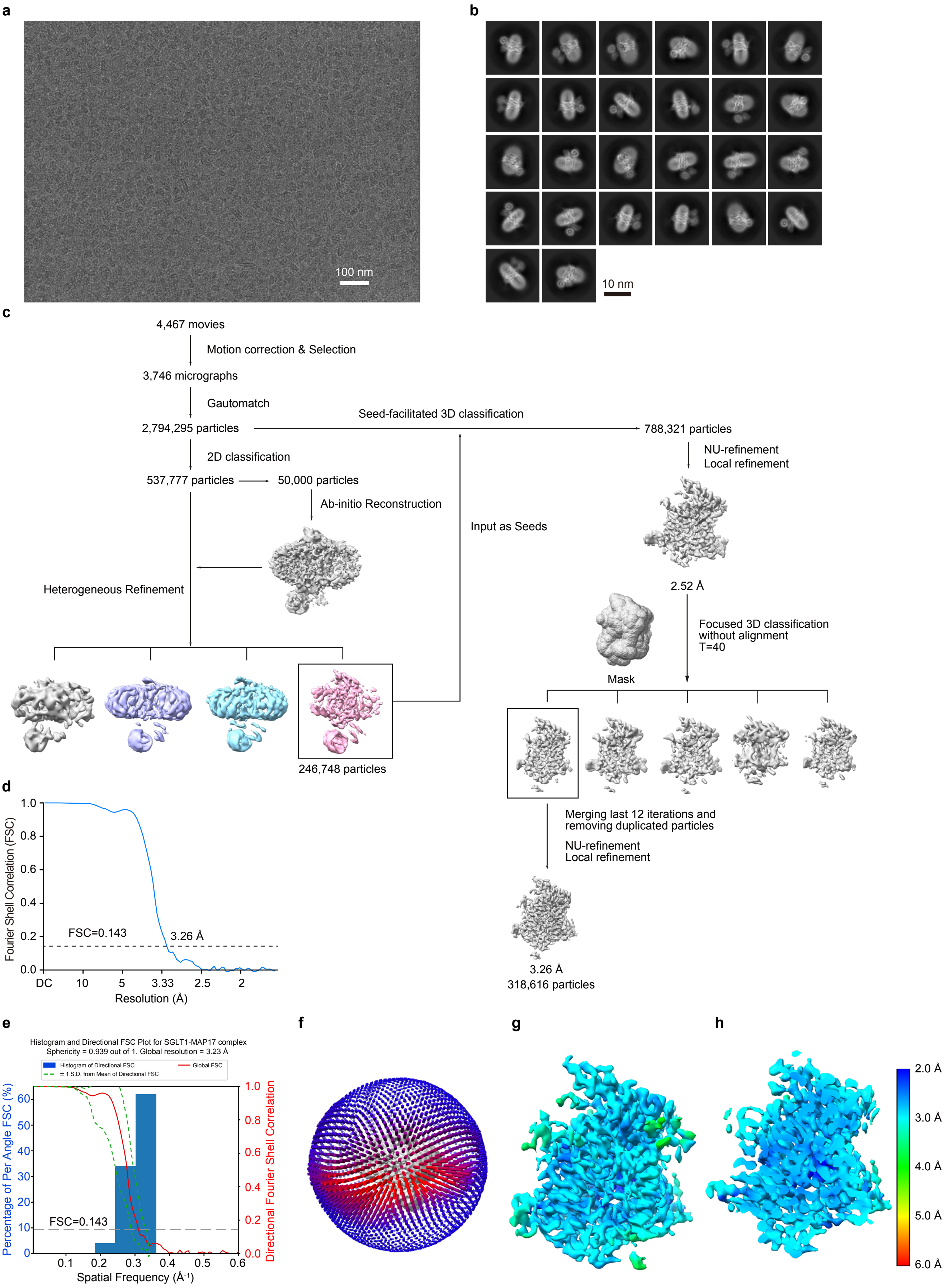

Supplementary Fig. 2 | Cryo-EM data processing of hSGLT1 in the 4D4FDG-bound state. a, Cryo-EM micrograph of hSGLT1. Experiment was repeated independently more than three times with similar results. **b**, 2D class averages of the particles used in the final reconstruction. **c**, Flowchart of image processing. **d**, The gold-standard FSC curve for the cryo-EM map. **e**, Histogram of directional FSC curves. Individual 1D FSC curves are compiled into 3D FSC and represented within a histogram. The spread of the directional resolutions defines by plus and minus one standard deviation from the mean of the directional resolutions. **f,** Euler angle distribution of all particle images that contributed to the final 3D map. **g**, Local resolution distribution of hSGLT1 estimated with cryoSPARC. **h,** The cut-open view of local resolution distribution of hSGLT1.

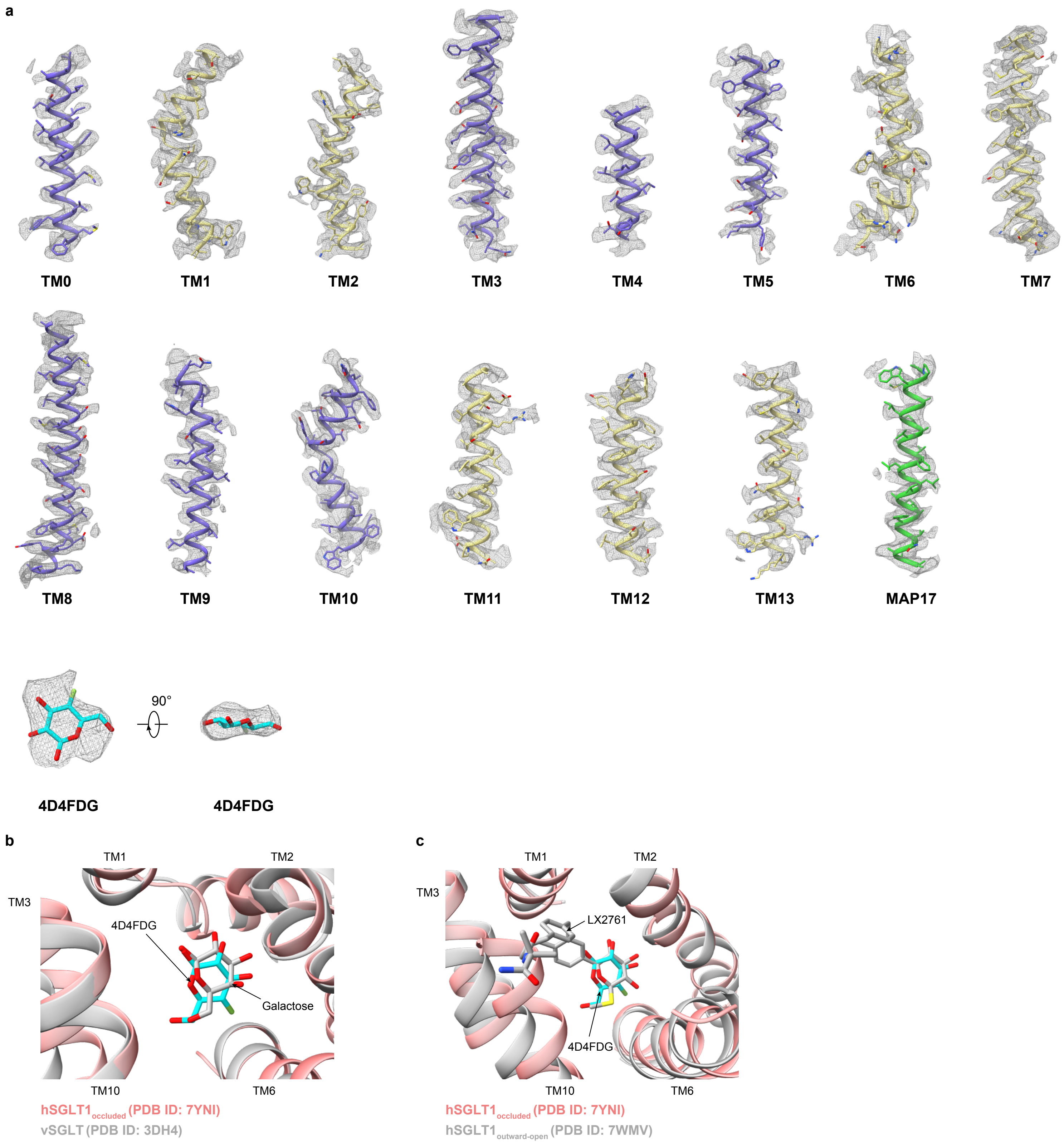

Supplementary Fig. 3 | Cryo-EM densities and structural models of hSGLT1. a, The PDB models of the hSGLT1-MAP17 complex are overlaid with the electron density shown in gray meshes. **b**, The structure of hSGLT1-4D4FDG (colored) was overlaid onto the vSGLT1-galactose structure (grey, PDB ID: 3DH4). Ligands were shown as sticks. **c**, The structure of the hSGLT1-4D4FDG (colored) was overlaid onto the hSGLT1-LX2761 structure (grey, PDB ID: 7WMV). Ligands were shown as sticks.

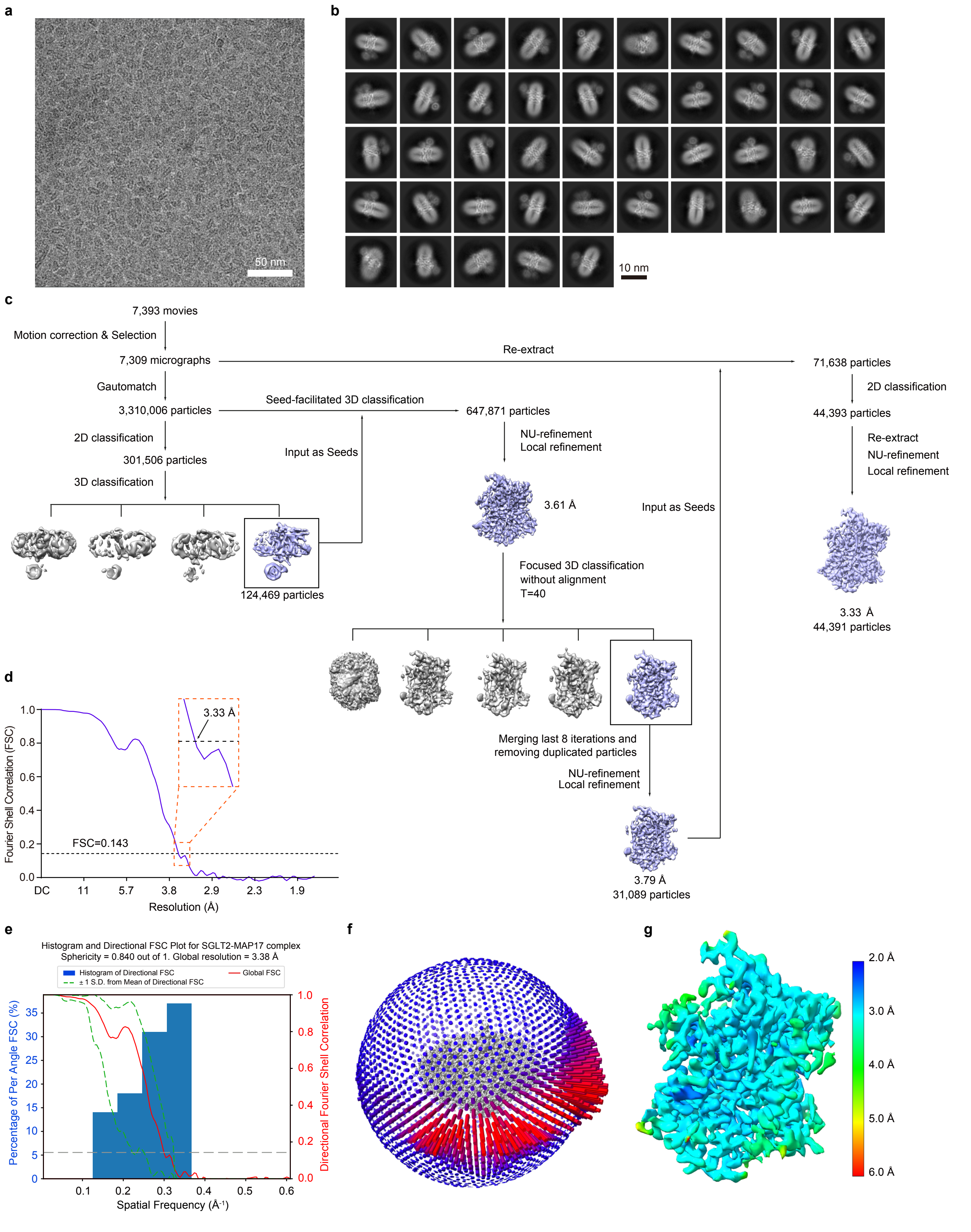

Supplementary Fig. 4 | Cryo-EM data processing of hSGLT2 in the AMG-bound state. a, Cryo-EM micrograph of hSGLT2. Experiment was repeated independently more than three times with similar results. **b**, 2D class averages of the particles used in the final reconstruction. **c**, Flowchart of image processing. **d**, The gold-standard FSC curve for the cryo-EM map. **e**, Histogram of directional FSC curves. Individual 1D FSC curves are compiled into 3D FSC and represented within a histogram. The spread of the directional resolutions defines by plus and minus one standard deviation from the mean of the directional resolutions. **f,** Euler angle distribution of all particle images that contributed to the final 3D map. **g**, Local resolution distribution of hSGLT2 estimated with cryoSPARC.

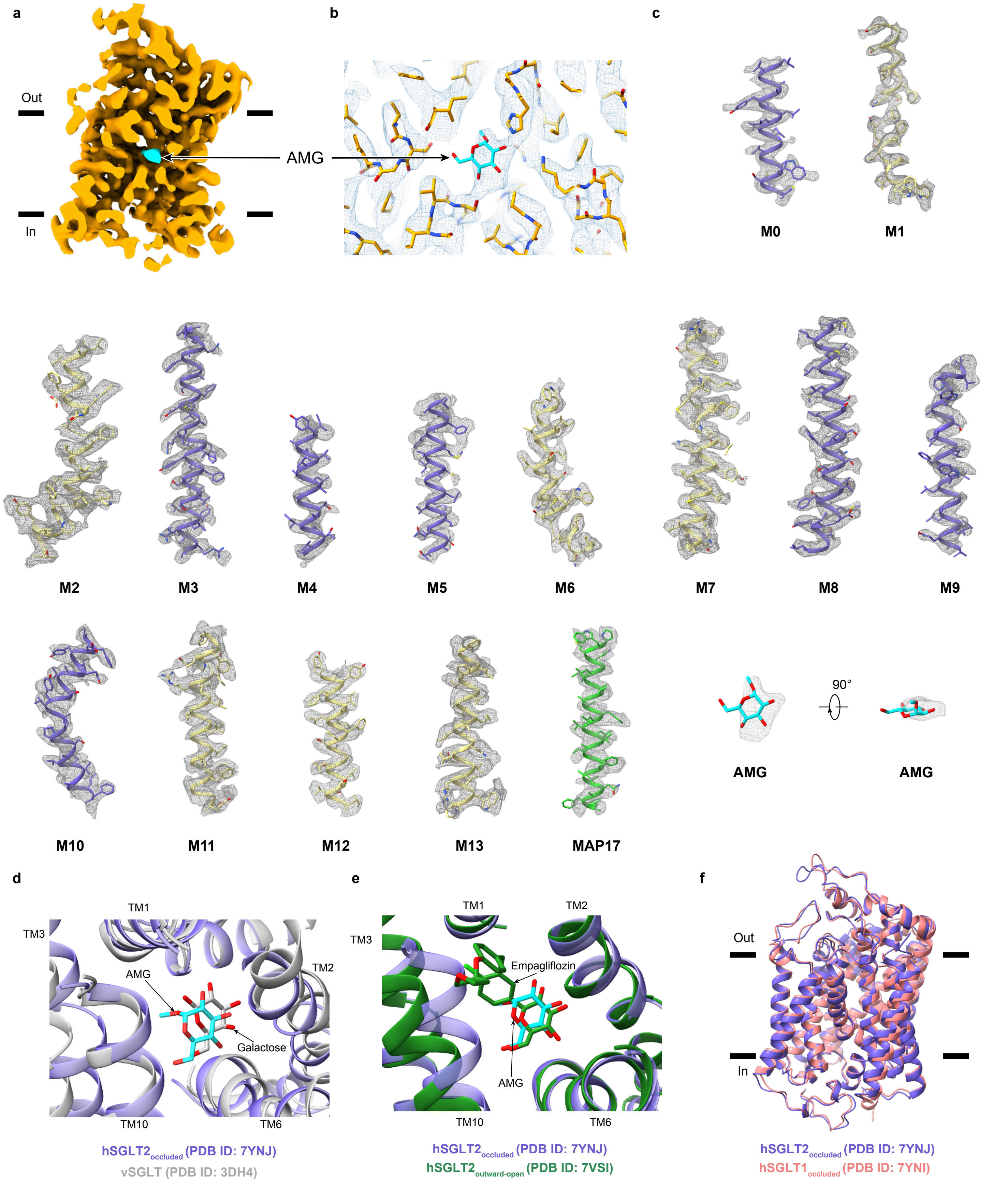

Supplementary Fig. 5 | Cryo-EM densities and structural model of the hSGLT2-MAP17 complex in the AMG-bound occluded state. a, The cut-open view of the hSGLT2-MAP17 complex shows the binding site of AMG substrate inside hSGLT2. AMG is colored in green. The hSGLT2 is colored in orange. **b**, The electron density of AMG and nearby residues. **c**, The PDB models of hSGLT2-MAP17 complex are overlaid with the electron density shown in gray meshes. **d**, The structure of hSGLT2 (colored) was overlaid onto the vSGLT1-galactose structure (grey, PDB ID: 3DH4). Ligands were shown as sticks. **e**, The structure of hSGLT2 (colored) was overlaid onto the hSGLT2-empagliflozin structure (grey, PDB ID: 7VSI). Ligands were shown as sticks. **f**, Structural superposition of $hSGLT1_{occluded}$ (pink) and $hSGLT2_{occluded}$ (purple).

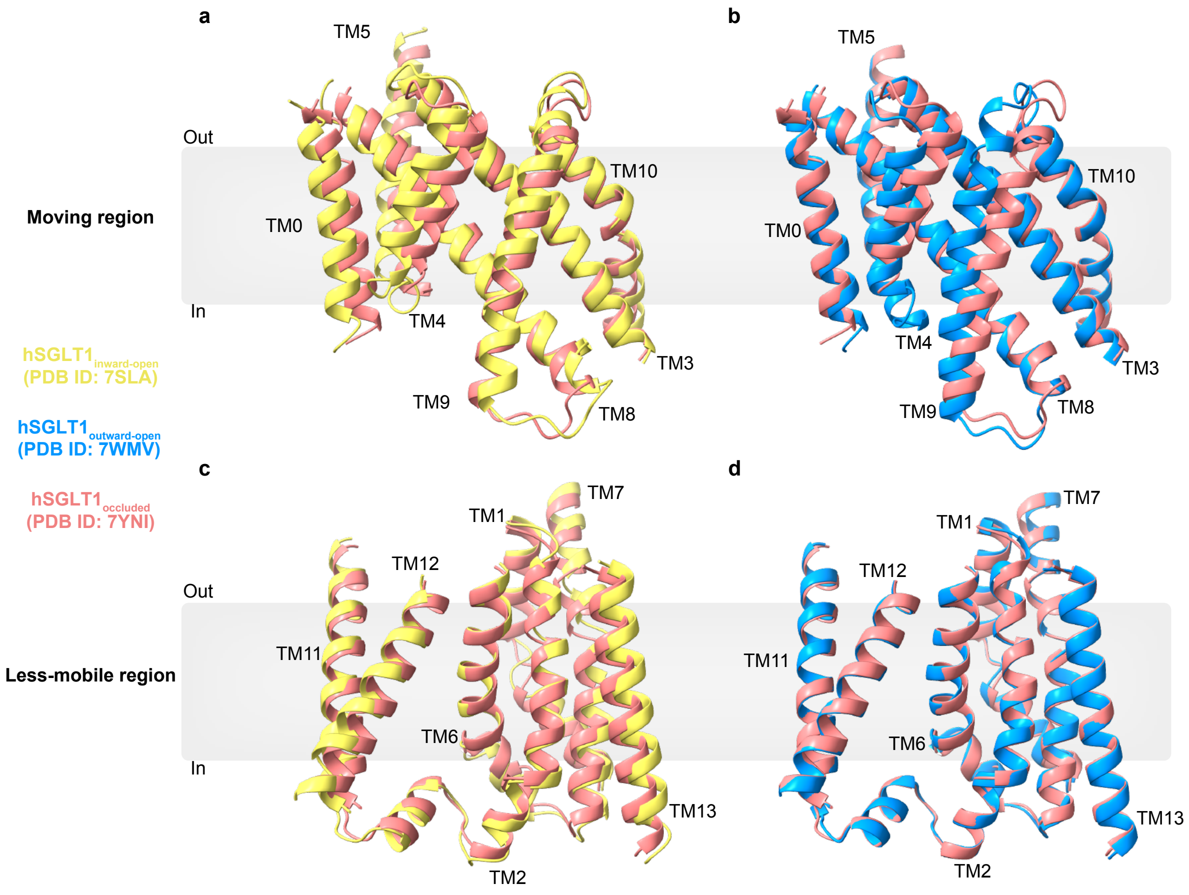

Supplementary Fig. 6 | Structural alignment of less-mobile region and moving region of hSGLT1. a, Superposition of the moving region of hSGLT1$_{\text{inward-open}}$ (yellow, PDB ID: 7SLA) and hSGLT1$_{\text{occluded}}$ (pink). **b,** Superposition of the moving region of hSGLT1$_{\text{outward-open}}$ (blue, PDB ID: 7WMV) and hSGLT1$_{\text{occluded}}$ (pink). **c,** Superposition of the less-mobile region of hSGLT1$_{\text{inward-open}}$ (yellow, PDB ID: 7SLA) and hSGLT1$_{\text{occluded}}$ (pink). **d,** Superposition of the less-mobile region of hSGLT1$_{\text{outward-open}}$ (blue, PDB ID: 7WMV) and hSGLT1$_{\text{occluded}}$ (pink).

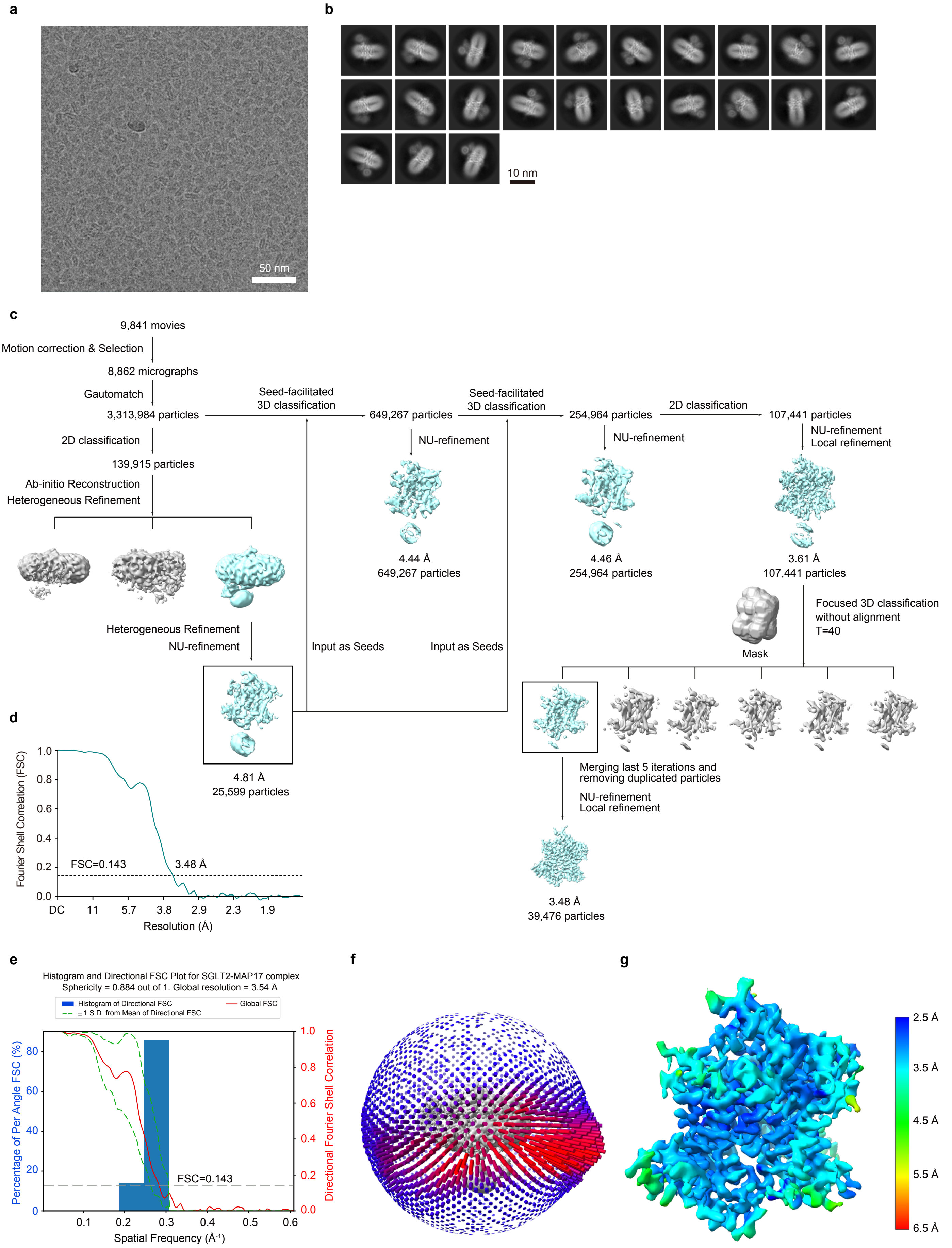

Supplementary Fig. 7 | Cryo-EM data processing of hSGLT2 in the apo state. **a**, Cryo-EM micrograph of hSGLT2. Experiment was repeated independently more than three times with similar results. **b**, 2D class averages of the particles used in the final reconstruction. **c**, Flowchart of image processing. **d**, The gold-standard FSC curve for the cryo-EM map. **e**, Histogram of directional FSC curves. Individual 1D FSC curves are compiled into the 3D FSC and represented within a histogram. The spread of the directional resolutions defined by plus and minus one standard deviation from the mean of the directional resolutions. **f,** Euler angle distribution of all particle images that contributed to the final 3D map. **g**, Local resolution distribution of hSGLT2 estimated with cryoSPARC.

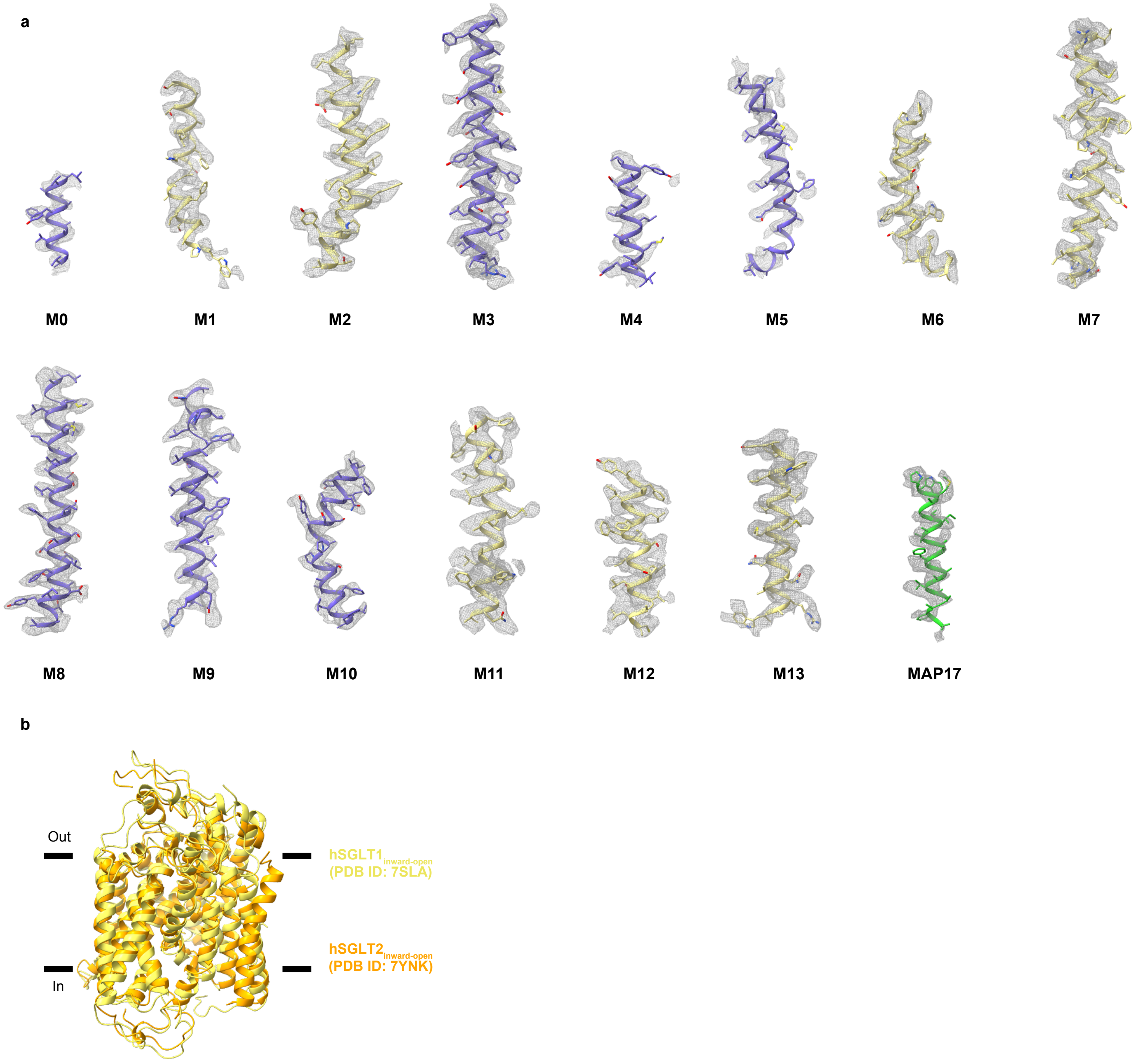

Supplementary Fig. 8 | Cryo-EM densities and structural model of the hSGLT2-MAP17 complex. a, The PDB models of the hSGLT2-MAP17 complex are overlaid with the electron density shown in gray meshes. **b,** Structural superposition of $hSGLT1_{inward\text{-}open}$ (yellow, PDB ID: 7SLA) and $hSGLT2_{inward\text{-}open}$ (orange).

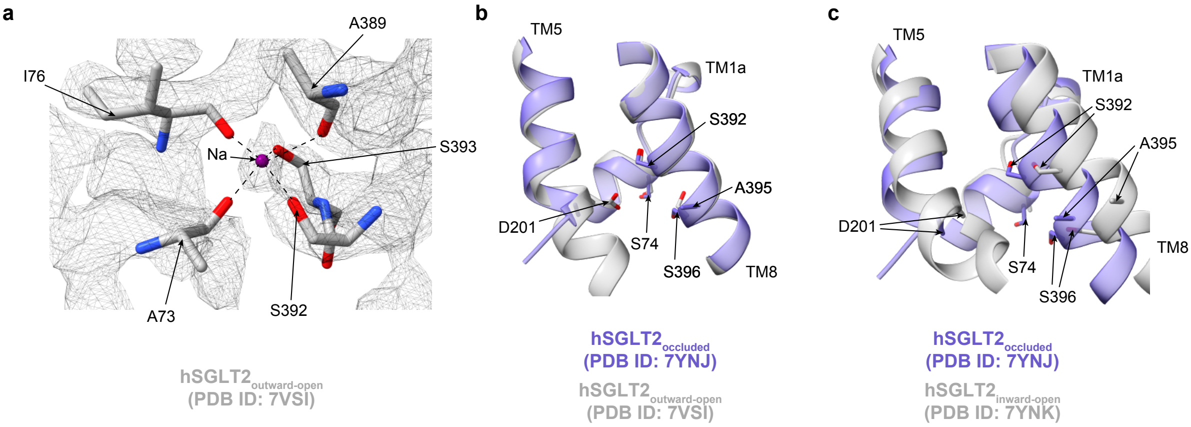

Supplementary Fig. 9 | Na binding sites in hSGLT2. a, The electron density map of the hSGLT2 Na2 site. The map of hSGLT2-empagliflozin (EMD-31558) was further sharpened with a B factor of -78.9 Å^2 in Coot to show the distinguished Na2 density and its binding groups. **b**, Structural comparison of the Na3 site of hSGLT2 between the outward-open (gray, PDB ID: 7VSI) and occluded (purple) states. **c**, Structural comparison of the Na3 site of hSGLT2 between the occluded (purple) and inward-open (gray) states.

Supplementary Table 1
Cryo-EM data collection, refinement and validation statistics

	hSGLT1-MAP17 complex with 4FG bound (EMD-33962) (PDB 7YNI)	hSGLT2-MAP17 complex with AMG bound (EMD-33963) (PDB 7YNJ)	hSGLT2-MAP17 complex (EMD-33964) (PDB 7YNK)
**Data collection and processing**			
Magnification	105,000×	165,000×	165,000×
Voltage (kV)	300	300	300
Electron exposure (e–/Å^2)	35.84	37.6	37.6
Defocus range (μm)	-1.5 to -1.8	-1.5 to -1.8	-1.5 to -1.8
Pixel size (Å)	0.834	0.821	0.821
Symmetry imposed	*C1*	*C1*	*C1*
Initial particle images (no.)	2,794,295	4,192,308	3,310,006
Final particle images (no.)	318,616	44,391	39,476
Map resolution (Å)	3.26	3.33	3.48
FSC threshold	0.143	0.143	0.143
Map resolution range (Å)	200-3.26	200-3.33	200-3.48
**Refinement**			
Initial model used (PDB code)	7WMV	7VSI	7VSI
Model resolution (Å)	3.26	3.33	3.48
FSC threshold	0.143	0.143	0.143
Model resolution range (Å)	200-3.26	200-3.33	200-3.48
Map sharpening *B* factor (Å^2)	-197.8	-131.7	-100
Model composition			
Non-hydrogen atoms	4387	4371	4123
Protein residues	594	592	578
Ligands	1	1	0
B factors (Å^2)			
Protein	81.73	58.94	45.89
Ligand	77.18	48.55	-
R.m.s. deviations			
Bond lengths (Å)	0.005	0.005	0.005
Bond angles (°)	1.045	1.047	1.055
Validation			
MolProbity score	2.16	1.88	1.87
Clashscore	19.14	13.14	10.53
Poor rotamers (%)	0.00	0.00	0.00
Ramachandran plot			
Favored (%)	94.50	96.22	95.26
Allowed (%)	4.98	3.78	4.74
Disallowed (%)	0.52	0.00	0.00
